# Office-Based Physical Activity and Nutrition Intervention: Barriers, Enablers, and Preferred Strategies for Workplace Obesity Prevention, Perth, Western Australia, 2012

**DOI:** 10.5888/pcd10.130029

**Published:** 2013-09-12

**Authors:** Krysten Blackford, Jonine Jancey, Peter Howat, Melissa Ledger, Andy H. Lee

**Affiliations:** Author Affiliations: Jonine Jancey, Andy H. Lee, School of Public Health, Curtin University, Bentley, Australia; Peter Howat, Centre for Behavioural Research in Cancer Control, Curtin University, Shenton Park, Australia; Melissa Ledger, Cancer Council Western Australia, Shenton Park, Australia.

## Abstract

**Introduction:**

Workplace health promotion programs to prevent overweight and obesity in office-based employees should be evidence-based and comprehensive and should consider behavioral, social, organizational, and environmental factors. The objective of this study was to identify barriers to and enablers of physical activity and nutrition as well as intervention strategies for health promotion in office-based workplaces in the Perth, Western Australia, metropolitan area in 2012.

**Methods:**

We conducted an online survey of 111 employees from 55 organizations. The online survey investigated demographics, individual and workplace characteristics, barriers and enablers, intervention-strategy preferences, and physical activity and nutrition behaviors. We used χ^2^ and Mann–Whitney *U* statistics to test for differences between age and sex groups for barriers and enablers, intervention-strategy preferences, and physical activity and nutrition behaviors. Stepwise multiple regression analysis determined factors that affect physical activity and nutrition behaviors.

**Results:**

We identified several factors that affected physical activity and nutrition behaviors, including the most common barriers (“too tired” and “access to unhealthy food”) and enablers (“enjoy physical activity” and “nutrition knowledge”). Intervention-strategy preferences demonstrated employee support for health promotion in the workplace.

**Conclusion:**

The findings provide useful insights into employees’ preferences for interventions; they can be used to develop comprehensive programs for evidence-based workplace health promotion that consider environmental and policy influences as well as the individual.

## Introduction

Poor nutrition and physical inactivity among employees affect the workplace; overweight and obesity are among the most common health issues affecting productivity (1). Physical inactivity has increased in workplaces because job roles have changed from manual labor to predominantly inactive tasks (2). Because of the common use of desktop computers, 45% of employed adults in Australia work in sedentary roles (3). Similarly, a rise in levels of automation and in the number of labor-saving devices has resulted in more people working in sedentary roles in the United States (4). The combination of sedentary behavior and easy access to energy-dense, nutrient-poor foods compounds the risk of overweight and obesity (4).

The workplace has a direct influence on employee health (5). For this reason, it is a critical setting for health promotion (2). Effective workplace health programs should incorporate behavioral, environmental, and policy approaches (6). It is therefore essential to have favorable physical and social environments as well as a supportive organizational culture to encourage positive behavior change (7).

Participation rates for workplace health programs vary among studies, demographic groups, and types of programs (8). Further investigation into barriers and enablers of program engagement among employees is needed, including examination of the characteristics of nonparticipating but eligible employees (9). Few studies describe effective workplace programs to prevent or manage overweight and obesity and reduce the burden of chronic disease. Evidence-based information for guiding the development of workplace health promotion programs is particularly lacking in Western Australia, the state with the highest prevalence (63%) of overweight/obesity in Australia (10).

The Social Cognitive Theory (11) provides the foundation for this study, addressing both underlying determinants of health behavior and methods of promoting change. This theory explores the importance of social norms and environmental influences on health behavior and how to modify these influences to achieve positive health outcomes (11). Exploring how employees perceive physical and nutrition and how they interact with their physical and social environment will identify barriers and enablers and strategies for workplace health promotion programs. The objective of this study was to identify barriers and enablers of physical activity and nutrition as well as intervention strategies (individual, environmental, policy) for health promotion in office-based workplaces in the Perth metropolitan area.

## Methods

We conducted an online survey among office-based employees in June 2012. For study inclusion, employees had to be aged 18 to 45 years, located in the Perth metropolitan area, and working in an office that had at least 20 staff members. The study received Curtin University Human Research Ethics approval (no. PH-71–2012).

In preparation for the survey, we conducted formative research in 2 steps. In March 2012, we conducted 4 focus groups with 37 employees. We recruited office-based workers, who represented a random sample of the target population, through a computer-assisted telephone interview system. The focus group discussion guide explored physical activity and nutrition behaviors, barriers and enablers to physical activity and healthy eating, and preferred intervention strategies for workplace health promotion. In April 2012, we conducted 15-minute semistructured telephone interviews of 10 managers from 8 organizations. The interviews explored managerial support for workplace health promotion. To participate, managers had to manage a team of at least 10 office-based staff in the Perth metropolitan area. We used professional networks and office-based worksites listed in the Perth telephone directory to recruit participants. 

We again used professional networks and office-based worksites listed in the Perth telephone directory to recruit participants for the online survey of office-based workers. We approached 410 employees; 137 employees representing 55 organizations agreed to participate. A link to the survey was sent to participants by e-mail after informal agreement to participate was established. After providing informed consent, participants completed the survey, which took approximately 10 to 15 minutes. Participants who had not commenced the survey within a week received a reminder e-mail.

The online survey (Appendix) collected information on participant demographic characteristics (sex, country of birth, home postcode, education, employment status, average number of hours worked per day, occupation, and year of birth) workplace characteristics (eg, industry type, number of employees, office location/postcode, and health-related activities offered), physical activity and nutrition barriers and enablers, physical activity and nutrition behaviors, preferred intervention strategies (activities, materials and resources, policies and environmental conditions) for workplace health promotion, and preferred methods of communication. Previous research (1,2), other scientific literature, and a validated instrument (12) helped the formulation of the questions. Extensive pilot testing preceded final implementation. Participants were placed in a drawing to win several $50 gift certificates.

We analyzed the quantitative data from the online survey by using SPSS version 20.0 (International Business Machines Corp, Armonk, New York). Participants were asked to answer questions on preferred intervention strategies by using a 5-point Likert scale. To facilitate analysis, answers to questions on intervention materials and resources were recoded into 3 groups: not preferred (strongly not preferred or not preferred), neutral (neutral), and preferred (preferred or strongly preferred). Answers to questions on physical activity and nutrition activities were recoded into the following: unlikely (very unlikely or unlikely), neutral (neutral), and likely (likely or very likely). Similarly, answers to questions on policy and environmental strategies were recoded into the following: disagree (strongly disagree or disagree), neutral (neutral), and agree (agree or strongly agree).

We quantified the number of participants in each category and used χ^2^ and Mann–Whitney *U* statistics to test for differences between age and sex groups for barriers and enablers, intervention-strategy preferences, and physical activity and nutrition behaviors. We divided the participants into 2 age groups: younger (18–31 y) and older (32–45 y).

Stepwise multiple regression analysis accounted for collinearity and determined factors affecting physical activity and nutrition behavior. The first step was to identify associations between independent variables (barriers and enablers, demographics, and individual and workplace characteristics) and dependent variables (total daily sitting time [hours per day], total daily walking time [minutes per day], total physical activity [minutes per day], and total fruit and vegetable consumption [grams per day]). Independent variables associated (*P* < .05) with the dependent variables were entered into the regression model.

## Results

Of 137 people who agreed to participate in the online survey, 111 (81.0%) completed it. The mean age of the respondents was 32.5 years; most were women (Table 1). Most respondents had a bachelor’s degree or higher, and most were employed full time. Two-thirds were Australian-born. Approximately 15% of respondents were insufficiently active (<30 min/d) and approximately half of respondents did not meet the recommended servings of fruits and vegetables per day. Most respondents worked in noncommercial industries (eg, education and training, government, health and medical, not-for-profit). Small- or medium-sized organizations (20–99 employees) employed more than half of the participants; the remainder worked in large organizations (≥100 employees).

**Table 1:** Demographic and Behavioral Characteristics of Office-Based Workers Who Participated in Online Survey (N = 111), Perth, Western Australia, 2012^a^

Characteristic	n (%)
**Sex**	
Male	24 (21.6)
Female	87 (78.4)
**Age**	
18–31 y	59 (53.2)
32–45 y	52 (46.8)
**Country of birth**	
Australia	75 (67.6)
Other	36 (32.4)
**Perth metropolitan postcode**	
North coastal	37 (33.3)
North inland/hills	24 (21.6)
South inland	23 (20.7)
South coastal	27 (24.3)
**Education level**	
Bachelor’s degree or higher	88 (79.3)
Trade, diploma, TAFE	13 (11.7)
Year 12 or equivalent	8 (7.2)
Year 10 or equivalent	2 (1.8)
**Employment status**	
Full time	89 (80.2)
Part time	12 (10.8)
Casual, contract, or temporary	10 (9.0)
**Sitting time, mean (SD), hours/day**	7.2 (1.8)
**Walking time, mean (SD), minutes/day**	36.8 (25.6)
**Total physical activity time, mean (SD), minutes/day**	58.1 (33.7)
**Fruits, mean (SD), servings/day**	2.6 (1.2)
**Vegetables, mean (SD), servings/day**	3.7 (1.3)

Abbreviations: TAFE, technical and further education; SD, standard deviation.

a Values are numbers (percentages) unless otherwise indicated.

### Barriers and enablers

The most common physical activity barriers were “too tired” (54.1%) and “work commitments/long hours” (53.2%). The older group was more likely than the younger group to cite family commitments as a barrier (53.8% vs 22.0%; *P* = .001). The most common physical activity enablers were “enjoy physical activity” (54.1%) and “health benefits” (54.1%). More men than women cited “being able to cycle/walk to shops/work” (54.2% vs 28.7%; *P* = .02), “shower and change rooms at work” (62.5% vs 25.3%; *P* = .001), and “work recreation teams” (25.0% vs 5.7%; *P* = .01).

The most common nutrition barriers were “unhealthy food available in office” (30.6%) and “lack of healthy options near office” (28.8%). The younger age group was more likely than the older group to cite the barriers “unhealthy food vending machines” (18.6% vs 3.8%; *P* = .02) and “healthy food more expensive than unhealthy food” (32.2% vs 15.4%; *P* = .04). The most common nutrition enablers included “food prepared at home” (84.7%) and “nutrition knowledge” (55.9%). We found no significant associations by age or sex.

### Behavior predictors

#### Total daily sitting time

For total daily sitting time, we found significant group differences according to 1 activity offered by the workplace (fitness assessments), 1 barrier (“work commitments/long hours”), and 1 enabler (“work recreation teams”). The regression model (Table 2) confirmed the association of these independent variables with total daily sitting time.

**Table 2:** Stepwise Regression Results for Physical Activity and Nutrition Behaviors for Office-Based Workers (N = 111), Perth, Western Australia, 2012

Variable	Unstandardized Coefficient (SE)	*P* Value
**Sitting time (hours/day)**		
Hours worked per day	0.37 (0.16)	.02
Work commitments/long hours	0.92 (0.33)	.006
Work recreation teams	−1.70 (0.52)	.001
**Walking time (minutes/day)**		
Lunch-time activities	14.94 (6.23)	.02
**Total physical activity time (minutes/day)**		
Cycle- or walk-to-work promotion	13.75 (5.89)	.02
Too tired	−11.53 (5.70)	.05
Family commitments	−19.88 (5.77)	.001
Health benefits	11.63 (5.65)	.04
Work recreation teams	25.08 (9.59)	.01
Male	15.53 (7.03)	.03
**Total fruit and vegetable consumption (grams/day)**		
No healthy food options near office	−103.86 (45.72)	.02
Food prepared at home	117.96 (58.39)	.05
Nutrition knowledge	87.28 (42.37)	.04

#### Total daily walking time

For total walking time, we found significant group differences according to 1 activity offered by the workplace (corporate gym membership) and 3 enablers (“colleagues to exercise with,” “lunch-time activities,” and “work recreation teams”). The regression model suggested that “lunch-time activities” is a significant predictor of total walking time.

#### Total daily physical activity time

For total daily physical activity time, we found significant group differences according to 1 demographic characteristic (sex), 2 activities offered by the workplace (health screening/assessments and a cycle- or walk-to-work promotion), 3 barriers (“too tired,” “family commitments,” and “lack of motivation/interest”); and 4 enablers (“enjoy physical activity,” “showers and change rooms at work,” “health benefits,” and “work recreation teams”). The regression model showed that sex, “work recreation teams” (an enabler), “work commitments/long hours” (a barrier), and “average number of hours worked per day” were significant predictors of total physical activity time.

#### Total fruit and vegetable consumption

For fruit and vegetable consumption, we found significant group differences according to 1 demographic characteristic (employment status); 1 barrier (“lack of healthy food options near office”); and 2 enablers (“food prepared at home” and “nutrition knowledge”). The regression model suggested that “nutrition knowledge” and “food prepared at home” (enablers) and “no healthy food options near office” (a barrier) are significant predictors of total fruit and vegetable consumption.

### Preferred intervention strategies

#### Physical activity

The most preferred activities were stretching program at desk (73.9%) and group classes (eg, yoga) (73.0%). More of the younger age group than the older age were likely to participate in a corporate gym membership (61.0% vs 40.8%, *P* = .01) and the Global Corporate Challenge (59.3% vs 36.2%, *P* = .05). A greater percentage of women than men stated they would be likely to participate in a walking group at lunch time (57.6% vs 26.1%, *P* = .02) and group classes (64.6% vs 26.1%, *P* = .004).

The preferred materials and resources were a stretching chart (72.1%) and a calendar (containing tips, information on topics such as goal setting) (70.2%). The younger age group had a greater preference for pedometers (70.2% vs 42.0%, *P* = .01), whereas women had a greater preference for pedometers (64.3% vs 30.4%, *P* = .01) and a calendar (58.3% vs 22.7%, *P* = 0.01).

The preferred policy/environmental supports were flexible work hours (94.6%) and shower and change facilities (90.1%). A greater percentage of women (36.9%) than men (14.3%) agreed that motivational posters reminding them to take the stairs would support them to be more active in the workplace (*P* = .04).

#### Nutrition

The preferred nutrition activities were personalized dietary programs (76.6%) and cooking demonstrations (73.0%). A greater percentage of the younger age group than the older age group were likely to participate in a weight-loss challenge with colleagues (58.6% vs 30.6%, *P* = .01). A greater percentage of women than men were likely to participate in cooking demonstrations (66.7% vs 33.3%, *P* = .006) and weight-loss challenges with colleagues (50.0% vs 28.6%, *P* = .003).

The preferred nutrition materials and resources were healthy recipes (83.8%) and a nutrition newsletter (79.3%). Women had a greater preference than men for a newsletter (70.9% vs 43.5%, *P* = .02), a healthy eating manual (70.2% vs 41.7%, *P* = .03), healthy recipes (77.6% vs 41.7%, *P* = .002), and a dietary guideline chart (54.1% vs 30.4%, *P* = .03).

The preferred policy/environmental supports were having a well-equipped kitchen at work (91.0%) and a free box of fruit provided in the kitchen (87.4%). A greater percentage of women (47.6%) than men (13.6%) agreed that motivational posters in the kitchen would support them to eat a healthful diet at work (*P* = .02).

### Preferred contact methods

The most preferred program delivery methods were e-mail (89.2%) and web-based/Internet (58.6%). The least preferred were telephone (1.8%) and mail (12.6%). A greater percentage of respondents from the younger age group preferred mail as a program delivery method (18.6% vs 5.8%, *P* = .04). The most preferred format was electronic (59.5%), and the least preferred was hard copy (5.4%).

## Discussion

This study identified several factors affecting physical activity and nutrition behaviors, including the most common barriers and enablers. Many barriers highlighted the limited amount of time available to employees outside of working hours and the need to ensure healthy and supportive environments in the workplace. Intervention-strategy preferences demonstrated employee support for health promotion in the workplace.

The reported barriers to physical activity and nutrition were quite different from each other —“too tired” and “work commitments/long hours” were the physical activity barriers most often cited. The most common barriers to eating a healthful diet were “unhealthy food available in office” and “lack of healthy options near office.” Studies on physical activity and nutrition have reported similar findings: “not enough time” and “work schedule” were identified as the most common physical activity barriers (13), and “lack of knowledge,” “lack of time,” and “presence of tempting and limited healthy food options” were the most common nutrition barriers cited (14). These findings highlight the challenge of long working hours and the responsibility of organizations to provide physical activity and nutrition programs to support employee health.

Because disparities exist in the level of physical activity among population subgroups (15), it is useful to identify enablers and predictors of physical activity behavior to better inform the development of workplace health interventions (16). Two barriers were significant predictors of physical activity in this study: employees who reported work commitments/long hours as a barrier reported 55 more minutes of total daily sitting time than those who did not cite this barrier, and employees who reported family commitments as a barrier participated in 19.9 fewer minutes of total daily physical activity than those who did not cite this barrier.

In contrast, employees participating in work recreation teams engaged in 25.1 more minutes of total daily physical activity. Similarly, those participating in lunch-time activities walked for 14.9 more minutes more per day than those who did not participate, and employees reporting “health benefits” as an enabler engaged in 11.6 more minutes of daily physical activity than those who did not report this enabler. These findings suggest that more emphasis should be placed on educating employees about the health benefits of physical activity.

Employees who indicated a lack of healthy food options near the office as a nutrition barrier reported consuming a smaller amount of fruit and vegetables per day (103.9 g) than those who did not report the barrier, whereas those who prepared food at home and had nutrition knowledge consumed more fruit (118.0 g) and vegetables (87.3 g) than those who did not prepare food at home or have nutrition knowledge. These findings indicate that employees working in a location that lacks healthy food options can be encouraged to prepare food at home and increase self-efficacy through nutrition education.

Participation in workplace health programs can vary among demographic groups and the types of programs offered (8). It is therefore important to identify preferences for individual, organizational, and policy intervention methods to enhance engagement and participation.

Preferences for physical activity strategies for the individual included stretching programs that can be performed at a desk, group classes, and the provision of pedometers, stretching charts, and calendars. The encouragement of light resistance training through stretching programs can provide multiple health benefits for employees who are normally sedentary and untrained (17). Group classes provide a socially supportive environment and an effective way to encourage physical activity (3).

More than half of respondents indicated a preference for pedometers. The use of pedometers can increase daily step counts and encourage physical activity during work hours (13); most staff members are usually willing to participate in activities using pedometers (18). Step counts can be increased by using pedometers in conjunction with diaries and self-monitoring methods such as a calendar (13).

The ability of environmental and policy strategies to increase physical activity in the workplace is well recognized (19). We found that among the most preferred workplace policies were flexible work hours. Breaks and flexible hours have a positive effect on employee physical activity and health (20).

Preferences for nutrition strategies for the individual were personalized dietary programs and cooking demonstrations, and the most preferred nutrition material/resource was healthy recipes. Such activities and resources are useful for improving knowledge, skills, and self-efficacy, which are significant mediators of increased fruit and vegetable intake (21).

Among the most preferred environmental and policy strategies were provision of fruit boxes and healthful food. A systematic review showed that environmental modifications affect the dietary intake of office-based employees (2). Strategies to improve dietary behaviors included ensuring availability of healthy food options, a finding consistent with other studies (22).

The most preferred method for information delivery was e-mail, followed by Internet-based delivery. Similarly, the most preferred format for program materials was electronic. The preference for electronic and Internet-based programs may be linked to the decreased stigma associated with enrolling in an Internet-based obesity program to address body weight (23). Electronic media have the advantage of having the capacity to reach large numbers of people at low cost (24).

Our study has several limitations. The survey recruitment process may have attracted more motivated individuals to the study (25). Also, most respondents were women; however, most employees at office-based worksites in Western Australia are women (10). Finally, the sample size (n = 111) may limit the generalizability of the study; nevertheless, such a sample size is deemed adequate for the purpose of formative research.

These findings can be generalized to most office-based worksites because we included numerous office sizes and industry types. Barriers and enablers provide information on factors to be overcome or encouraged through health promotion programs. Preferences for intervention strategies indicate strategies that are most and least likely to engage employees. In addition, our methods and survey instrument can serve as models for formative evaluation to assist development of office-based obesity prevention in other regions.

Findings from this study increase current understanding of the barriers, enablers, and preferred intervention strategies for programs to prevent obesity in office-based employees. Workplace health programs should be designed and implemented to ensure optimal reach and to provide benefits to employees and their organizations (26). Our findings can be used to develop comprehensive evidence-based workplace health promotion programs that consider the individual as well as environmental and policy influences (16).

## Appendix. Text of Online Survey Questions and Response Options

**Section 1: Information About You**

1. Sex: male, female
2. Country of birth: Australia, other.
3. Home postcode: [fill-in-the-blank].
4. Highest level of education: left school before year 10; year 10 or equivalent; year 12 or equivalent; trade, diploma or TAFE [technical or further education], bachelor degree or higher.
5. Employment status: full time, part time, casual/contract/temp.
6. Hours worked per day (average): 1 or less, 2, 3, 4, 5, 6, 7, 8, 9, 10+.
7. Occupation: [fill-in-the-blank].
8. Year of birth [fill-in-the-blank].

**Section 2: Information About Your Workplace**

1. Industry: building and construction, education and training, entertainment, financial services, tourism and hospitality, government, health and medical, mining and energy, not-for-profit, retail and sales, transport, other [fill-in-the-blank].
2. Approximately how many staff work in your office/workplace? Only include the number of people located in your office building, and not the total number for your whole organisation if located across a number of different sites: [fill-in-the-blank].
3. Office location/postcode: [fill-in-the-blank].
4. What health related activities does your workplace currently offer? Please select all that apply: allocated stretching/relaxing times; flexible work hours; corporate sports teams; physical activity sessions; health screening/assessments; fitness assessments; sports days/activities; corporate gym membership; cycle or walk to work promotion; sponsored events, eg, fun runs; group classes, eg, yoga; personalised diet programs; healthy cooking demonstrations; fruit and vegetable boxes; nutrition awareness/education; none; unsure; other [fill-in-the blank].

**Section 3: Barriers and Enablers**

1. Which of the following currently *limit* you being able to undertake regular *physical activity* during a *typical work day* (choose all that apply)? None; too tired; work commitments/long hours; family commitments; weather; lack of motivation/interest; financial cost; health reasons; unsure of the benefits of physical activity; lack of access to facilities/activities; time to commute to and from work; no workplace support; getting hot and sweaty; other [fill-in-the-blank].
2. Which of the following currently *enable or encourage* you to undertake regular *physical activity* during a *typical work day* (choose all that apply)? None; being able to cycle/walk to shops/work; enjoy physical activity; flexible work hours; colleagues to exercise with; challenges/competitions; weather; showers and change rooms at work; workplace support; health benefits; lunch-time activities; corporate gym membership; work recreation teams; other [fill-in-the-blank].
3. Which of the following currently *limit* your ability to consume a *healthy diet* during a *typical work day* (choose all that apply)? None; family commitments; lack of equipped kitchen at work; work commitments/long hours; lack of healthy food options near office; unhealthy food vending machines; unhealthy food available in office (fundraisers, morning teas, etc); healthy food more expensive than unhealthy food; my lack of cooking skills; my lack of nutrition knowledge; other [fill-in-the-blank].
4. Which of the following currently *enable or encourage* you to consume a *healthy diet* during a *typical work day* (choose all that apply)? None; healthy food supplied at office; access to healthy food outlets; food prepared at home; healthy food vending machines; kitchen facilities at work; colleagues eat healthy food; cooking skills; nutrition knowledge; other [fill-in-the-blank].

**Section 4: Preferred Physical Activity Strategies**

We are now going to ask you about your preferences for *activities* to support/encourage your participation in workplace *physical activity*.

1. Indicate how likely you would be to participate in the following physical activities if offered in your workplace [scale of 1 (very unlikely) to 5 (very likely)]: jogging/running group at lunch time; walking group at lunch time; team sports at lunch time; Global Corporate Challenge (eg, pedometer challenge); corporate gym membership; sponsored events, eg, fun runs; group classes, eg, yoga, Pilates; stretching program at desk; 2 options for other [fill-in-the-blank].

We are now going to ask you about your preference for materials and *resources* to support/encourage your participation in workplace *physical activity*.

2. Indicate which of the following physical activity materials and resources you would prefer in your workplace (if free) [scale of 1 (not preferred) to 5 (preferred)]: pedometer; newsletter (physical activity tips, health benefits, exercises, etc); stretching chart; calendar (events, physical activity tips, goal setting, etc); physical activity booklet (stretches, exercises, tips, goal setting, etc); health alerts via text message; health alerts via email; web-based physical activity information/materials; 2 options for other [fill-in-the-blank].

We are now going to ask you about *policy and environmental* strategies to support/encourage your participation in workplace *physical activity*.

3. Indicate whether you agree or disagree with the following statements [scale of 1 (strongly disagree) to 5 (strongly agree)]. The following do/would support me to be physically active in the workplace: flexible work hours; extended lunch breaks; incentives (eg, small prizes and gifts); 10 min breaks every 2 hours; shower and change facilities; facilities such as bicycle racks; motivational posters reminding me to take the stairs instead of the lift; easy access to stairs; 2 options for other [fill-in-the-blank].

**Section 5: Preferred Nutrition Strategies**

We are now going to ask you about your preference for *activities* to support/encourage *healthy eating* in the workplace.

1. Indicate how likely you would be to participate in the following nutrition activities if offered in your workplace [scale of 1 (very unlikely) to 5 (very likely): nutrition education sessions; veggie garden competitions; cooking demonstrations; personalised dietary program; weight loss challenge with colleagues; 2 options for other [fill-in-the-blank].

We are now going to ask you about your preference for *materials & resources* to support/encourage *healthy eating* in the workplace.

2. Indicate which of the following nutrition materials you would prefer to receive in the workplace: newsletter containing recipes, tips, etc; healthy eating manual containing recipes, nutrition information, health tips, etc; healthy recipes; dietary guideline chart outlining food groups and how much to eat; nutrition alerts via text message; nutrition alerts via e-mail; web-based nutrition information; 2 options for other [fill-in-the-blank].

We are now going to ask you about *policy and environmental* strategies to support/encourage your *healthy eating* in the workplace.

3. Indicate whether you agree or disagree with the following statements [scale of 1 (strongly disagree) to 5 (strongly agree). The following do/would support me to eat a healthy diet in the workplace: free cooking sessions; free nutrition education sessions; healthy food catering policy (healthy options at meetings, morning teas, etc); fruit boxes; healthy eating posters in kitchen/meals areas; healthy options in vending machines; well-equipped kitchen at work (eg, microwave, toaster, fridge, etc); 2 options for other [fill-in-the-blank].

**Section 6: Preferred Contact Methods**

1. If you were to participate in a workplace health promotion program, how would you like the program information to be delivered? (Tick all that apply): SMS; e-mail; phone; mail; web-based/Internet; online forums/discussions; health seminars; none; other [fill-in-the-blank].
2. Preferred format of program materials (e.g. newsletters, manuals etc): hard copy, electronic; both.
3. How often would you like to participate/receive updates? I would not participate; weekly; fortnightly; monthly; other [fill-in-the-blank].

**Section 7: Physical Activity**

The next section will ask you about your *physical activity* during a typical work day.

1. During a *typical work day*, how many hours do you spend *sitting*? 1 hour; 2 hours; 3 hours; 4 hours; 5 hours; 6 hours; 7 hours; 8 hours; 9 hours; 10 hours; 11 hours; 12 hours; 13+ hours.
2. During a *typical work day*, how many minutes do you spend *walking for transport?* (Examples include walking to/from work, walking to shops etc): [fill-in-the blank].
3. During a *typical work day*, how many minutes do you spend *walking for recreation*? (Examples include brisk walking during lunch or before/after work): [fill-in-the blank].
4. During a *typical work day*, how many minutes do you spend engaging in *moderate/vigorous physical activity*? (Examples include swimming, cycling, squash, jogging, circuit training, rowing, etc): [fill-in-the blank].
  - What moderate/vigorous activities you do? None; team sports (football, netball, etc); circuit training; jogging; cycling; rowing; swimming; other [fill-in-the blank].

**Section 8: Nutrition**

The next section will ask you about your *nutrition* during a typical work day.

1. How many * serves of fruit* do you eat in a * typical work day*? [Line drawing of an apple, 2 apricots, and 1 cup of chopped fruit answers question, “What is a serve of fruit?” Additional text says, “One serve equals 1 medium piece (an apple, banana, orange or pear); 2 small pieces (apricots, plums, or kiwi fruit); 1 cup diced pieces or canned fruit; ½ cup 100% juice; 1½ tablespoons dried sultanas or 4 dried apricots.”] Indicate the number of serves consumed per day on average: 0, 1, 2, 3, 4, 5, 6, 7+.
2. How many *serves of vegetables* do you eat in a *typical work day*? [Line drawing of ½ cup chopped vegetables or legumes, 1 medium potato, and 1 cup salad vegetables answer question, “What is a serve of vegetables?” Additional text says, “One serve equals 1 cup of salad, 1 medium potato, ½ cup of cooked vegetables; ½ cup of cooked, dried or canned beans, peas or lentils.” Indicate the number of serves consumed per day on average: 0, 1, 2, 3, 4, 5, 6, 7+.
